# Research on the Characteristics and Mechanism of the Cumulative Release of Antimony from an Antimony Smelting Slag Stacking Area under Rainfall Leaching

**DOI:** 10.1155/2017/7206876

**Published:** 2017-07-18

**Authors:** Bozhi Ren, Yingying Zhou, Andrew S. Hursthouse, Renjian Deng

**Affiliations:** ^1^Hunan Provincial Key Laboratory of Shale Gas Resource Exploitation, Hunan University of Science and Technology, Xiangtan 411201, China; ^2^School of Science & Sport, University of the West of Scotland, Paisley PA1 2BE, UK

## Abstract

We aimed to study the characteristics and the mechanism of the cumulative release of antimony at an antimony smelting slag stacking area in southern China. A series of dynamic and static leaching experiments to simulate the effects of rainfall were carried out. The results showed that the release of antimony from smelting slag increased with a decrease in the solid-liquid ratio, and the maximum accumulated release was found to be 42.13 mg Sb/kg waste and 34.26 mg Sb/kg waste with a solid/liquid ratio of 1 : 20; the maximum amount of antimony was released within 149–420 *μ*m size fraction with 7.09 mg/L of the cumulative leaching. Also, the antimony release was the greatest and most rapid at pH 7.0 with the minimum release found at pH 4.0. With an increase in rainfall duration, the antimony release increased. The influence of variation in rainfall intensity on the release of antimony from smelting slag was small.

## 1. Introduction

Antimony (Sb) is a typical toxic and harmful heavy metal element; the United Nations health organization stipulates that the body's daily intake of antimony in water should be less than 0.86 mg/kg [1, 2]. However, with the exploitation and utilization of antimony ore resources, a large amount of antimony slag is produced from antimony smelting and perennially exposed on the surface in a number of regions in China. Under the continuous action of rain, the process of desorption and mass transfer results in the introduction of antimony from the solid waste into mining area and its surrounding water environment, creating regional water environment pollution problems with great risk to human health [3–6]. Recent studies have concentrated on the release of heavy metal elements in soil, sludge, dust, and other solid media and the influence of a range of factors on waste leaching patterns [5, 7–14]. However, few reports exist on the release characteristics and cumulative leaching mechanisms of antimony in smelting slag stacking area under sustained rainfall. This study, therefore, evaluated a release test on antimony smelting waste slag with simulated rainfall. Guo et al. [5] comprehensively investigated the physical and chemical characteristics of the leaching of the solid wastes as from some core metal production areas. The more hazardous materials were identified. Hu et al. [15] studied the leaching characteristics and changes in the leached layer of antimony-bearing ores from China. The sources of release of some hazardous elements such as Sb, As, Pb, and Cr from typical antimony ores were determined. More and more studies [16–18] have been undertaken to provide better understanding of behavior of metallic elements in ore area. However, few studies have assessed the release and cumulative leaching characteristics of antimony from waste in smelting slag stacking area under sustained rainfall. Therefore, using simulated rainfall, test experiments on the release of antimony from smelting waste slag were conducted.

The study investigated the cumulative leaching release characteristics of antimony smelting slag, the impact of particle size, solid-liquid ratio, pH, the effects of the rainfall intensity, and rainfall duration. These characteristics were investigated to establish a dynamic model for antimony release. The model application is anticipated to assist in the understanding of the prevention and treatment of contamination from antimony and other nonferrous metal mining area, also to promote sustainable long-term development of nonferrous metal mining area.

## 2. Materials and Methods

### 2.1. Test Material

Test samples were collected from the open piles of smelting slag in the south ore smelting plant of Lengshui River, Hunan, China. Sampling was carried out to ensure representative material was collected, taking into account the different slag pile density, humidity, and antimony content. The collected samples were disaggregated before screening with ore (2 mesh sieve) before drying to provide uniform particle size. A simulated rainfall leaching solution was generated using deionized water, with pH control using a volume ratio of 6 : 1 (H2SO4 and HNO3) for acid-rain simulation, using NaOH solution to adjust for alkaline conditions.

### 2.2. Test Apparatus and Method

#### 2.2.1. Static Immersion Test

**Figure 1:**
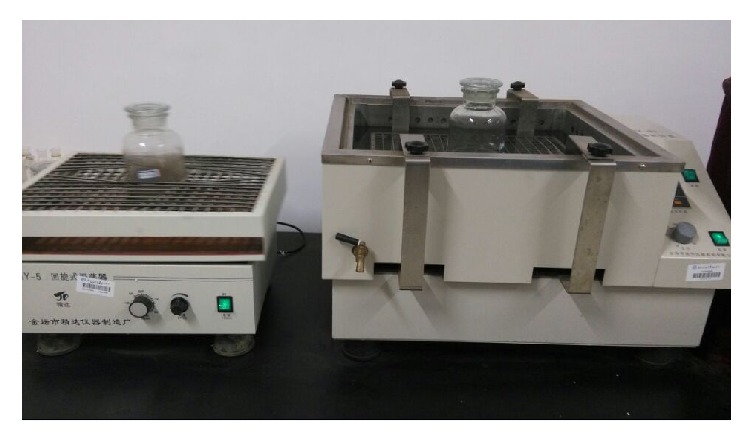
Experiment device of static leaching.

Static immersion test device consists of a constant temperature shaking bath and the wide-mouth bottle, shown in Figure 1. The shaking bath ensures constant temperature and minimizes external disturbance.

1. Immersion test for different solid/liquid ratios: samples for immersion tests were with solid/liquid ratios (w/v) of 1 : 5, 1 : 10, and 1 : 20, respectively, using 500 mL deionized water as the soaking solution, with pH adjusted to 4.0 to meet local acid-rain conditions. In addition, the test was maintained at 25 degrees in a constant temperature shaking bath for 12 days. Daily access to the supernatant was carried out to remove solution, filtered with 0.45 *μ*m membrane, and analyzed for antimony.
2. Immersion test in various particle sizes: selected tailings, blast furnace slag, and mining waste rock samples of 100 g were of size ranges within 420–841 *μ*m, 149–420 *μ*m, and <149 *μ*m, and the solid-liquid ratio is 1 : 5. The experiments were carried out as described above (1).

#### 2.2.2. Simulated Rainfall Leaching Test

**Figure 2:**
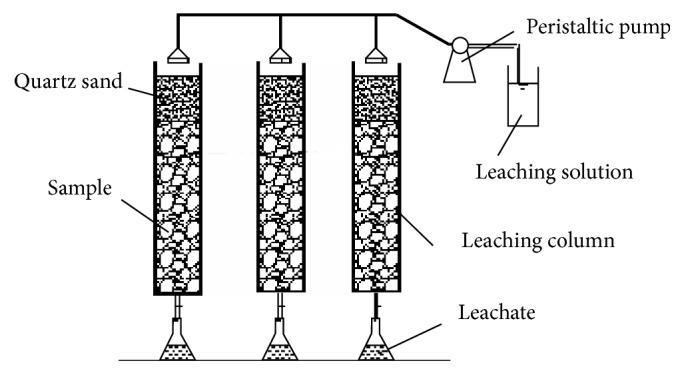
Schematic diagram of the leaching test.

The dynamic leaching test device for simulated rainfall consists of dynamic leaching column, peristaltic pump (supplying solution), and sampling bottle. The dynamic leaching column used a series of graduated glass columns to synchronize the leaching test, shown in Figure 2. Dimensions were as follows: radius 5 cm, length 50 cm of leaching column, and sealed 2-3 *μ*m pore diameter nonwoven glass sheets with a hole Φ10 mm at the bottom of the column to prevent sample loss. Inside the leaching column, from top to bottom in turn were two layers of ashless filter paper, clean quartz sand to 5 cm depth (to ensure uniform water distribution), and then the test sample.

1. Leaching test in different rainfall pH: a bulk sample of 600 g of each material was divided into three leaching columns and parallel control groups for each waste were established. The columns were subject to inverse saturation with water before the beginning of the experiment to ensure the test conditions are consistent. Regulated leaching by solutions of pH 2.0, 4.0, 5.6, 7.0, and 8.0 was performed separately. The daily amount of leaching was 600 ml that was carried out for a total of 12 days. The release of antimony in each waste and leachate conductivity over time was measured using standard tests.
2. Leaching experiment with different rainfall durations: in order to distinguish the effect of different rainfall duration on antimony release, a dynamic leaching column was set up with a constant irrigation speed (100 ml/h), the average daily amount of leaching converted from the yearly average precipitation is 648 ml/day. Leaching was carried out for 4, 6, 8, and 12 hours at constant intensity.
3. Leaching experiment with different rainfall intensity: the control of the flow rate for the peristaltic pump was used to explore the impact of rainfall intensity on antimony release. The daily volume of leaching solution used was consistent with other experiments (the daily annual average rainfall 684 mL). The rotational speed of peristaltic pump was adjusted to 40 mL·h^−1^, 60 mL·h^−1^, 80 mL·h^−1^, and 100 mL·h^−1^, corresponding to realistic actual rainfall simulation of light rain, moderate rain, heavy rain, and rainstorm.

### 2.3. Analytical Methods

The antimony concentration in leachate was determined by atomic absorption spectroscopy (ZEEnit700, Analytic Jena) using suitable calibration standards and reagent blanks; the pH value was determined using PB-10 (Sartorius) and the total solution conductivity was determined using portable conductivity meter (DDBJ-350, Nanbei). Daily leaching concentration of antimony per unit mass of waste sample was calculated using the following formula: *A* = (*Q* × *C*)/*M*, where *A* is daily precipitation concentration (mg/Kg); *Q* is the intraday addition of leaching solution (mL); *C* is daily leachate concentration of antimony (mg/L); and *M* is the weight of waste (Kg).

## 3. Results and Discussion

### 3.1. The Precipitation Characteristics of Antimony in Different Solid-Liquid Ratio and Particle Size

**Figure 3:**
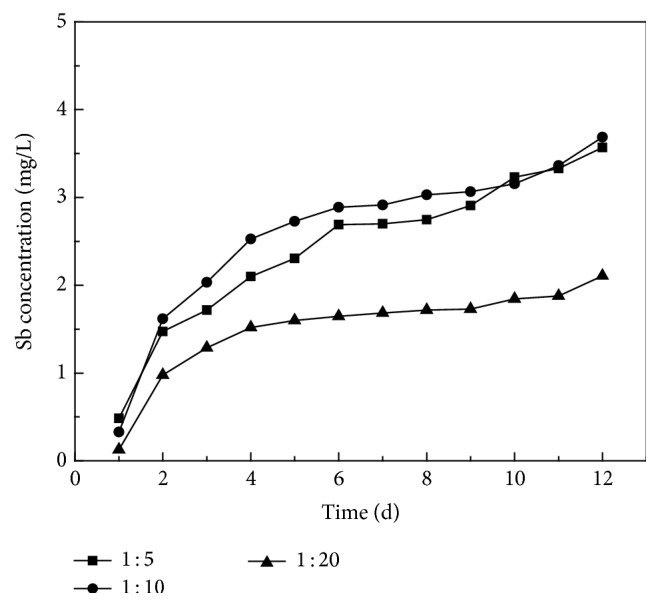
Effect of solid-to-liquid ratio on Sb release characteristic of smelting slag in static immersion test.

From Figure 3, the trends in the release of antimony from smelting slag under different solid-solution ratios were clearly separate. The maximum release concentration from the slag was at the beginning of the leaching period when the initial solid-liquid ratio was 1 : 10 and with continued immersion reaching a maximum converging with the results for solid-solution ratio of 1 : 5. In all cases, the overall trend that is leaching at the beginning of the test is relatively fast, and the rate gradually decreases with time.

**Figure 4:**
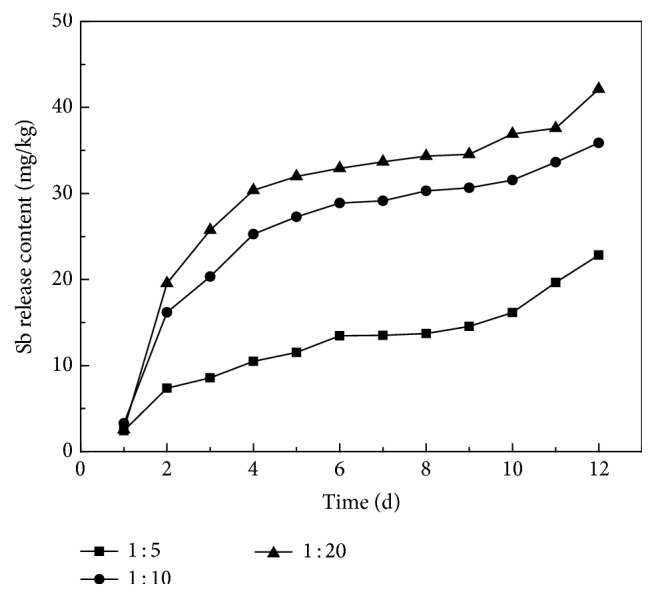
Sb release content per unit weight waste of smelting slag in static immersion test.

The conversion of this data to the amount of dissolution per unit mass at the various solid-liquid ratios (Figure 4 and Table 1) indicated that, during the static immersion, the release of antimony from the blast furnace slag increases gradually with the decrease of the ratio of solid to liquid. The maximum amount of antimony released at the end of trial is 42.13 mg/Kg. This confirms the effect of increased precipitation in the waste deposits.

**Figure 5:**
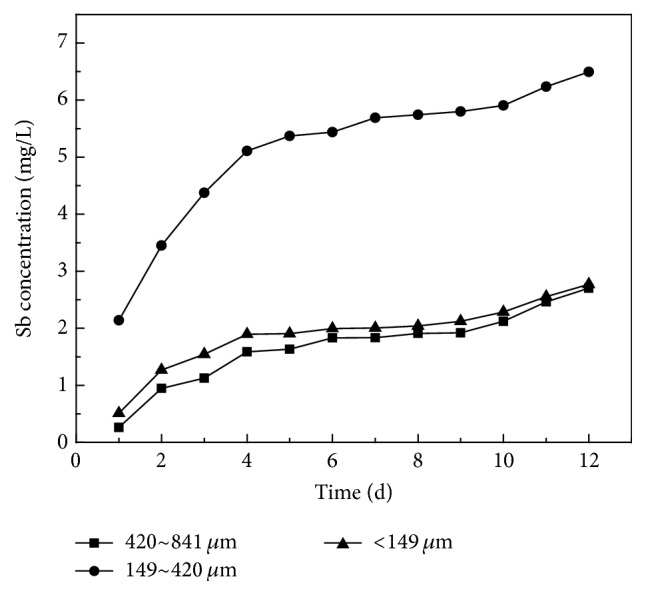
Effect of particle size on Sb release characteristic of smelting slag in static immersion test.

The effect of particle size variation on leaching of antimony (Figure 5) shows that, for the 149–420 *μ*m, size range in the highest level was obtained. The cumulative concentration in the cycle was 7.09 mg/L, while the release was significantly less for other particle sizes. The main influencing factors on the response to static immersion are hydrolysis oxidation reactions on the waste surface. With the decrease in particle size, the specific surface area and the reaction contact area increase, which make the reaction rate accelerate so that the amount released increases. Thus, the amount released for particle size 149–841 *μ*m is greater than that of the size range 420–841 *μ*m. However, when the waste particle size is less than 149 *μ*m, the amount released falls instead of rising. The explanation is that the particle size, despite the increase of specific surface area, decreases the contact area between leaching solution and air, inhibiting the oxidation process.

### 3.2. The Effect of pH Value in Rainfall

**Figure 6:**
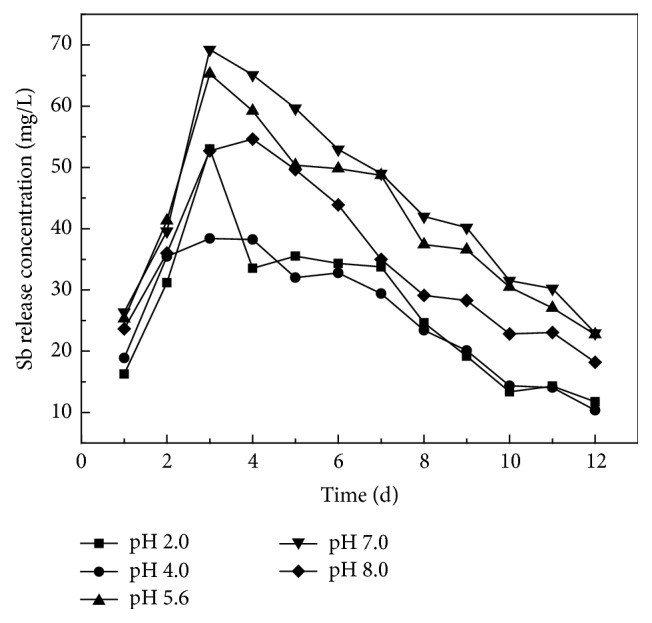
Sb concentration in leachate.

From Figure 6, the highest amount released and fastest release rate for antimony in smelting slag were when the leaching pH was 7.0, occurring at the third day and a total daily maximum leached concentration of 69.24 mg/L. Overall the release decreased in order of pH 5.6, 2.0, and 8.0, with the minimum at pH 4.0, with a daily maximum released concentration of 38.41 mg/L. A study of the reactions during antimony smelting in the Lengshuijiang River region shows the process of antimony smelting is divided into two steps: smelting of crude antimony and refining. The smelting of crude antimony requires a high temperature environment to allow the oxidation reaction of antimony sulfide and subsequent desulfurization [5].

In this way, the antimony oxide containing arsenic, lead, and other impurities (namely, crude antimony) is created and to remove the arsenic impurity, further refining is required. At present, the method of removing arsenic in the process of smelting antimony is mainly an alkaline air oxidation at high temperature. The addition of soda ash (Na_2_CO_3_) and introducing air during crude antimony smelting allow arsenic to oxidize, which is separated from antimony to produce sodium arsenate. Thus the composition of antimony smelting slag is chemically complex and may contain the antimony oxide losses and antimony compounds produced by redox reactions in the process of smelting under alkaline conditions.

Through leaching in surface environments, the antimony compounds release antimony through hydrolysis. But under weak acid or alkali conditions, the hydrolysis of antimony can be inhibited, reducing the leaching of antimony. If the strength of weak acid is relatively high, it neutralizes alkaline content of the antimony compounds and can accelerate the hydrolysis process, leaching more antimony. As shown in Figure 6, no matter what pH value is used, the daily amount leached always increases initially and then decreases. The maximum amount released always appears in third day, which shows that pH has little effect on the speed of antimony leaching but does impact on the amount released.

### 3.3. The Variation Characteristics of pH Value and EC Value in the Leachate

**Figure 7:**
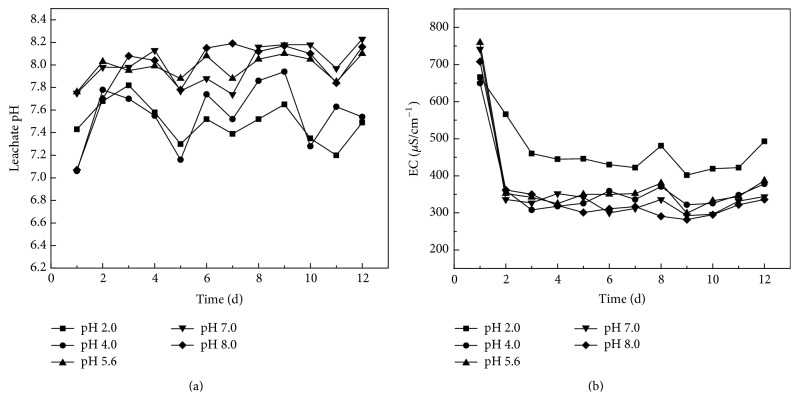
Change of pH and electroconductivity in leachate.

From Figure 7, the pH value and conductivity value (EC) during the leaching process show a fluctuation around neutral to partial alkalinity during the daily release process. This is due to the fact that adding alkali to remove arsenic during refining will release caustic dross containing antimony [15]. As seen in Figure 7(a) the results from leaching at pH 2.0 and pH 4.0 are lower than that of pH 5, pH 6, pH 7.0, and pH 8.0, which highlights the neutralization and buffering of the waste material, lowering the amount of antimony leached (Figure 6).

The leachate conductivity should reflect the total electrolyte activity and highlight the degree of ion release in the leaching process. As seen from Figure 7(b), it is obvious that the EC value is higher when the study starts, but the EC value decreases rapidly in the following two days and then stabilizes in a range with an extremely small fluctuation. The fast initial reaction stage releases various metal ions attached to the mineral surface, base cations in the smelting process and acid-base ions, and any precipitate from the slag itself. The conductivity of leaching solution gradually stabilizes with the time due to exchange reaction equilibrium.

### 3.4. The Impact of Rainfall Duration on Influencing the Release of Antimony in Leachate

**Figure 8:**
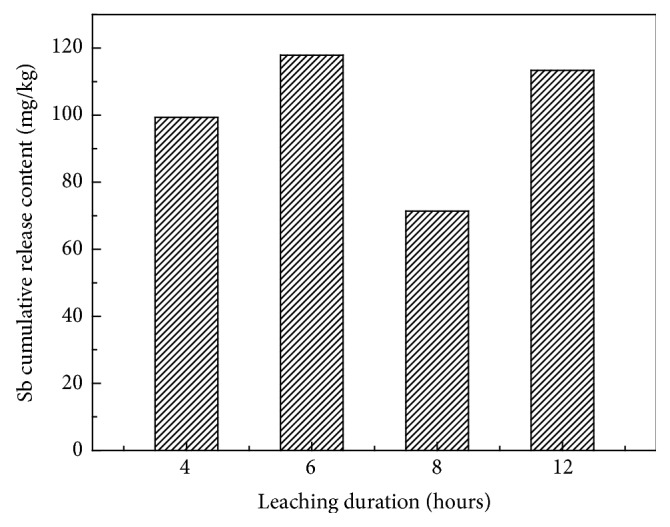
Cumulative amount under different rainfall duration.

The cumulative release of antimony (Sb) during the leaching process was assessed as shown in Table 2 and Figure 8. From Table 2, the daily release and accumulation of antimony are unequal due to the different leaching times (rainfall duration) each day as the rate and solution are constant. The release of antimony shows an overall increasing trend which increases to a maximum and then decreases with the leaching time (duration) increasing. The maximum cumulative release is 117.92 mg·kg^−1^ for 6 hours of leaching time (duration), and the minimum is 71.36 mg·kg^−1^ when the leaching time continued to 8 hours. The effect of reduced leaching as duration increases relates to the need for further oxidation in the slag for hydrolysis to release solid phase antimony.

The decrease in leaching may also be due to the surface pores of slag becoming eroded, leading to surface collapse and reduction of the contact area between slag, water, and air. At longer leaching time, the slag may have become more strongly eroded and further contact between water and air allows access to mineral surface. Thus more antimony is oxidized by water and air increasing the cumulative leaching. The precipitation profile of climate in southern China is likely to show increasing leaching and risk to the water environment from waste leaching.

### 3.5. The Effect of Rainfall Intensity on Influencing the Release of Antimony in Leachate

The higher the rainfall intensity, the stronger the physical and chemical erosion of waste in stacking area, particularly for surface waste deposits. However, the higher rainfall intensity with shorter duration and the same level of rainfall means the leaching time and oxidation release will be reduced. Therefore, it is important to analyze the influence of intensity on the leaching of waste in a fixed rainfall event.

**Figure 9:**
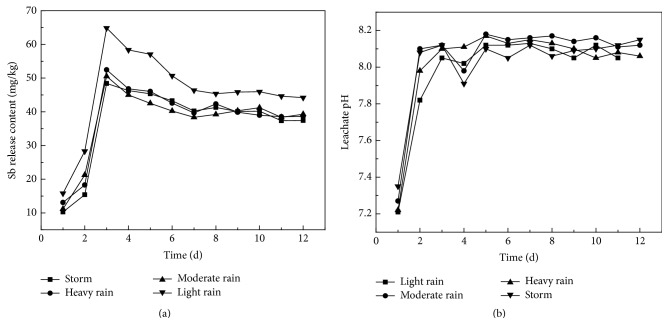
Change of pH and release amount of Sb in different rainfall intensity.

Figure 9(a) illustrates the impact of different rain intensity with the same weight of smelting slag and the total amount of rainfall. When the rainfall is light, the release of antimony is higher than in the other three types of rainfall, and leaching rate for antimony is quicker. This phenomenon suggests that the main factor of releasing the heavy metal is the oxidation of the metal phase, water and air access to the surface waste area, rather than scouring action of runoff. As seen from Figure 9(b), regardless of intensity in leaching, the pH value is always maintained around pH 8.1 except for the first day in the cycle, which suggests again that the reaction oxidizes antimony containing alkaline salts, resulting in that the pH value of leachate is generated as weakly alkaline.

### 3.6. TEM Analysis of Smelting Slag in Antimony

**Figure 10:**
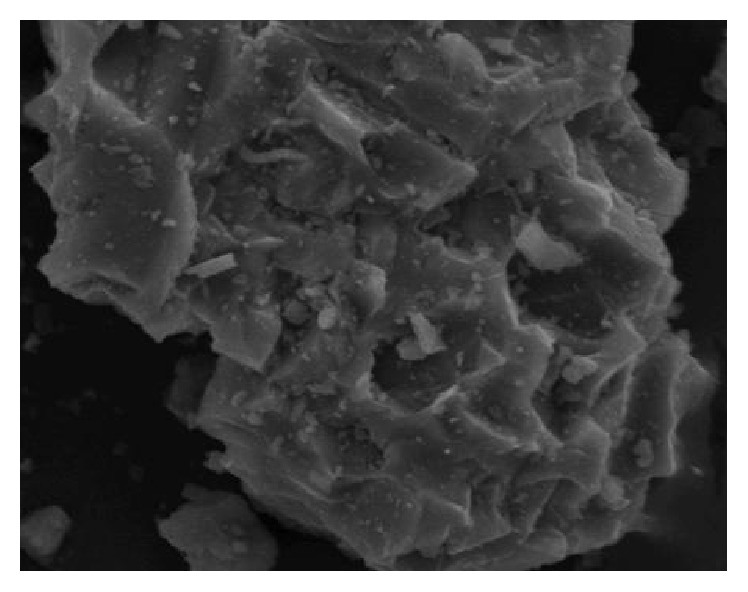
Result of SEM of antimony ore smelting slag.

The antimony ore smelting slag at 2000 times magnification effect of TEM analysis is shown in Figure 10. It can be seen that the surface of smelting slag from antimony ore is rough and contains many pores formed during the smelting process. These can also increase the contact area between water and air and the waste, enhancing the progression of oxidation-reduction reactions.

### 3.7. Mechanism Analysis of Rainfall Leaching Process

Through the outlined experimental approach, with the exclusion of the involvement of microbial activity, evidence is provided for the process of leaching of smelting slag.

*(1) Physical Reaction.* At the beginning of process, smelting slag exposed on the surface covered by soluble minerals and salts is washed out immediately into the leachate hit by the shear force of rain. The concentration of antimony increases rapidly.

*(2) Chemical Reaction.* With continued rainfall, soluble minerals adsorbed on the solid surface are washed away; hence waste internally begins, in contact with rain, to release antimony through oxidation on the surface.

Meanwhile, the alkali ions inside the waste exchange with surface oxidation simultaneously, causing the liquid phase to become alkaline. In this connection, various microgalvanic cell reactions occur in the spaces between a variety of ore slag particles and particles inside with leaching of antimony, increasing the concentration to the maximum [16].

**Figure 11:**
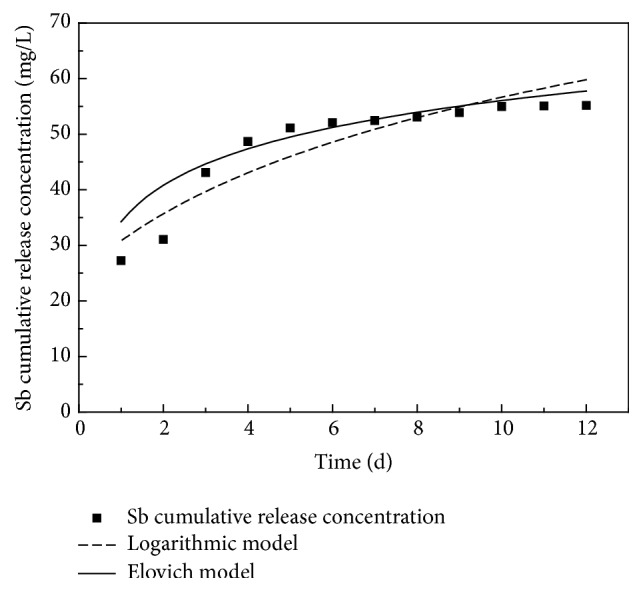
Data fitting of the Sb release with Elovich model.

The principle of the release of antimony from smelting slag under the process of rainfall leaching can be studied using the release kinetics model of Elovich, widely used in soil chemical kinetics research [13]. The model can describe the release mechanism of smelting slag under rainfall erosion, verified by simulation of acid rain from leaching column experiment. From Figure 11, the Elovich model appears a good fit for the process of cumulative release. The parameters derived from this process are shown in Table 3.

## 4. Conclusion

Through static and dynamic experiments, the study on the cumulative release characteristics and mechanism of antimony in leaching from ore processing slag can be summarized as follows:

1. The trends of antimony release are affected by soli-liquid ratios; the lower solid-liquid ratio is accompanied by higher released concentration with greater leached amount of per mass unit of waste and the proportion of dissolution; the maximum amount of antimony released was from the 149~420 *μ*m particle size range with 7.09 mg/L cumulative leaching concentration. It is one of reasons for increased concentrations following increasing rainfall in stacking area of antimony smelting slag.
2. The released amount and its rate are the greatest for pH value of rainfall in neutral and weakly basic conditions. The change of pH value of the solution had no effect on the release trend. The process of leaching by rainfall could be divided into two steps: the initial stage of surface erosion and the later stage of internal oxidation according to analyzing the changes of pH value and EC value. The leaching amount and dissolution rate of antimony from smelting slag reached the maximum when pH value is at 7.
3. The difference in rainfall duration could bring about diverse impacts on the effects of slag on the wider environment. The trend in cumulative release increases before decreasing and starts to grow. Except for light rain, the effect of rainfall intensity on antimony release is relatively low.
4. The process of leaching consists of both physical and chemical reactions. The study has highlighted the Elovich model as being useful in describing leaching release kinetics of smelting slag, with good fit for the process of cumulative release.

## Figures and Tables

**Table 1:** Accumulation release concentration and dissolution rate of smelting slag.

Solid/liquid ratio	Smelting slag	
	Cumulative release content (mg/kg)	Dissolution ratio (%)
1 : 5	22.850	0.029
1 : 10	35.875	0.046
1 : 20	42.132	0.054

**Table 2:** Cumulative release amount of Sb in smelting slag under different rainfall duration.

Duration (h)	Sb release content (mg/kg)												
	1	2	3	4	5	6	7	8	9	10	11	12	∑∑
4.0	6.33	7.36	8.15	7.86	10.09	9.18	7.13	8.25	9.32	10.23	8.25	7.23	99.35
6.0	8.62	10.49	13.07	13.81	16.30	15.11	13.57	12.36	14.59	0	0	0	117.92
8.0	11.80	8.74	7.50	7.22	12.52	10.00	13.58	0	0	0	0	0	71.36
12.0	19.64	20.56	23.96	23.56	25.63	0	0	0	0	0	0	0	113.35

**Table 3:** Fitting information with logarithmic and Elovich model.

	*A*	*B*	*R* ^2^
Logarithmic model	−3.04	22.06	0.82
Elovich model	34.24	9.47	0.92
